# Spatial Quality Evaluation of Resampled Unmanned Aerial Vehicle-Imagery for Weed Mapping

**DOI:** 10.3390/s150819688

**Published:** 2015-08-12

**Authors:** Irene Borra-Serrano, José Manuel Peña, Jorge Torres-Sánchez, Francisco Javier Mesas-Carrascosa, Francisca López-Granados

**Affiliations:** 1 Institute for Sustainable Agriculture, IAS-CSIC, P.O. Box 4084, Córdoba 14080, Spain; E-Mails: jmpena@ias.csic.es (J.M.P.); jtorres@ias.csic.es (J.T.-S.); flgranados@ias.csic.es (F.L.-G.); 2 Department of Graphic Engineering and Geomatics, Campus de Rabanales, University of Cordoba, Córdoba 14071, Spain; E-Mail: ig2mecaf@uco.es

**Keywords:** UAV, ortho-mosaicked image, resampling, OBIA, weed mapping, visible (RGB), near-infrared (NIR)

## Abstract

Unmanned aerial vehicles (UAVs) combined with different spectral range sensors are an emerging technology for providing early weed maps for optimizing herbicide applications. Considering that weeds, at very early phenological stages, are similar spectrally and in appearance, three major components are relevant: spatial resolution, type of sensor and classification algorithm. Resampling is a technique to create a new version of an image with a different width and/or height in pixels, and it has been used in satellite imagery with different spatial and temporal resolutions. In this paper, the efficiency of resampled-images (RS-images) created from real UAV-images (UAV-images; the UAVs were equipped with two types of sensors, *i.e.*, visible and visible plus near-infrared spectra) captured at different altitudes is examined to test the quality of the RS-image output. The performance of the object-based-image-analysis (OBIA) implemented for the early weed mapping using different weed thresholds was also evaluated. Our results showed that resampling accurately extracted the spectral values from high spatial resolution UAV-images at an altitude of 30 m and the RS-image data at altitudes of 60 and 100 m, was able to provide accurate weed cover and herbicide application maps compared with UAV-images from real flights.

## 1. Introduction

Even when the patchy distribution of weeds in sunflower fields, as well as the variability in the abundance and type of weeds, has been demonstrated using on-ground sampling [1,2,3], herbicides are usually broadcast over the entire field, including weed-free zones, using a unique kind of herbicide. This extensive application of herbicides has not only relevant economic and environmental implications, but also plays a role in the development of herbicide-resistant weed biotypes [4,5,6,7].

To overcome this situation, site-specific weed management (SSWM) could be used as an alternative to adjust the herbicide treatment to the weed patches only and to consider different herbicide applications according to the weed species, weed group composition (e.g., against broadleaved, grass or resistant weeds) or weed thresholds (*i.e.*, the weed infestation cover above which a treatment is required) [8,9,10,11]. Moreover, recent findings have led to a changing perception on weed thresholds in agro-ecosystems. The main findings are that some groups of weeds have numerous beneficial interactions with other organisms (e.g., pollinators) or are important to maintain biodiversity, and some of these interactions can have direct effects on the functioning of the agro-ecosystem [12]. A combination of SSWM according to the weed threshold would provide efficient weed control allowing both biodiversity provision and crop production. Therefore, an effective strategy may consist of the use of a single herbicide treatment for weed patches where a unique group of weeds are present, the use of several herbicides depending on the presence of different weed species or group compositions, or the use of a herbicide treatment based on percentage weed cover or weed threshold.

To make this possible, remotely sensed imagery from satellite or piloted aircraft have successfully been used to create accurate weed maps at flowering or late phenological stages to facilitate decision making and reach the objective of SSWM [13,14,15,16,17]. However, the use of these remote platforms for mapping weeds within crops at very early phenological stages is limited due to their coarse image spatial resolution (usually >50 cm pixel). This is not sufficient to distinguish between weeds and crop species, because of their small size and spectral appearance similarities at that early stage [18]. Unmanned aerial vehicles (UAVs) are emerging as an appropriate technology to collect the images required for this task [19,20]. The UAVs can fly at very low altitudes to generate fine spatial and temporal resolution imagery (flights can be programmed on demand depending on the objective of each study), their acquisition costs are low, and different sensors with diverse spectral ranges can be embedded. These characteristics facilitate the procurement of high spatial, spectral and temporal resolutions, which are required for the agronomic goal of detecting weeds at early stages [21].

However, similar to most technology, the UAVs have some limitations and technical problems, e.g., stabilization may not be constant at high flight altitudes (e.g., 100 m) due to wind being more noticeable, the battery determines the duration of the flight and flight altitude is restricted to 120 m by the Spanish regulation for UAVs <25 kg. This affects the pixel size and dimensions of the surface covered by each flight because the lower the flight altitude, the higher the spatial resolution but the lower the surface coverage. Consequently, a set of overlapping images is required to cover the whole study area. These images must be stitched together to create an ortho-mosaicked image that requires aerotriangulation and ortho-rectification, which are time-consuming processes. This is facilitated by a number of invariant features (generally present in any scenario) and geo-locating targets (Ground Control Points, GCP), which are placed in the field and ultimately used in a geo-registration process to determine the spatial quality of the mosaicked image [22]. These steps become more complicated for real crop scenarios, such as herbaceous row crops that are flown over because of their repetitive pattern and the difficulty in identifying invariant or specific features.

Resampling is a mathematical technique that is used to create a new version of a remotely sensed image with a different width and/or height in pixels, *i.e.*, a process that geometrically transforms digital images [23,24]. Increasing the size of an image (making the pixel size smaller and consequently increasing both the number of pixels of the original image and the spatial resolution) is called upsampling, whereas reducing the size of an image (larger pixel size but fewer number of pixels and lower spatial resolution) is called downsampling [25]. Digital image resampling originated in the early 1970s [23] and has mostly been used for improving the amount of information that can be extracted from satellite imagery with a coarse spatial and fine temporal resolution (such as NOAA-AVHRR: 1.1 km spatial resolution and 1–3 images per day, or TERRA-MODIS: spatial resolution of 250 m and one image every two days), and medium spatial but low temporal resolution (e.g., Landsat 7 ETM: 30 m spatial resolution, one image every 16 days) [26,27,28]. The objective of these works was to combine the advantageous characteristics of every sensor, *i.e.*, the Landsat 7 ETM spatial information and the temporal frequency of NOAA-AVHRR or TERRA-MODIS data. The resulting RS-image must have high quality to ensure the accuracy of the numerical and visual output. In the case of working to resample a UAV-image, the objective would be to create an RS-image to simulate higher flight altitudes with a corresponding larger pixel size and lower number of pixels by using the UAV-images obtained at a low altitude with a higher spatial resolution (*i.e.*, lower pixel size) and a high number of pixels. That is, this resampling will allow a flight at low altitude to be used as a baseline (high spatial resolution) to obtain a new image at a higher altitude (lower spatial resolution) without carrying out the real flight at a high altitude. This will reduce the personnel, economic and time resources involved with the flights. In addition, the data post-processing and image analysis can be optimized (due to a lower number of pixels), which avoids new image ortho-rectification and mosaicking because these two procedures would already have been conducted for the low altitude flight or for flight altitude optimizing for a specific objective in scientific studies.

Object-based image analysis (OBIA) is a powerful procedure and an efficient and accurate alternative to pixel-based methods [29,30,31,32] to discriminate crops, bare soil and early weeds, and can be used to generate weed maps. The OBIA approach first identifies spatially and spectrally homogeneous units (objects) created by grouping adjacent pixels according to a procedure known as segmentation. Then, automated and auto-adaptive classification methods are developed using the objects as the minimum information units, and their spectral, contextual (position, orientation), morphological and hierarchical information is combined. Peña *et al.* [33] used the OBIA strategy for early-season weed discrimination in maize using non-mosaicked UAV-imagery and a three-step automatic classification approach focused on crop line detection. However, it would be necessary to obtain the weed patch information over the whole crop field using an ortho-mosaicked image to facilitate a complete georeferenced map of weed cover for the SSWM equipment.

Taking into account the factors introduced above, the objectives of this work were to: (1) resample imagery from UAVs at different flight altitudes and evaluate the similarity and quality of the resulting RS-images based on visual and mathematical criteria; (2) apply an OBIA procedure for crop and weed patch detection in RS-images and UAV-images; (3) evaluate and compare the weed map outputs obtained from UAV-images *vs.* RS-images by establishing different weed thresholds; and (4) evaluate and discuss the resampling limitations and opportunities that are related to optimizing the UAV technology and time-consuming processes under several potential scenarios.

## 2. Experimental Section

### 2.1. Locations, Flights and Sensors

The study was conducted in two sunflower fields (named Fields 1 and 2) located in Monclova farm (province of Seville, southern Spain) of approximately 1 ha each. The flights were authorized by a written agreement between the farm owners and our research group. The geographic coordinates (UTM, zone 30N, WGS-84) of the upper left corner of the images were X = 295,400 m Y = 4,156,107 m, and X = 295,112 m Y = 4,155,611 m, respectively. Both sites were naturally infested by broadleaved weeds such as *Amaranthus blitoides S. Wats*, *Sinapis arvensis L.*, *Convolvulus arvensis L*. and *Chenopodium album L*. The vegetative growth stage of weeds and crop in both fields were in the principal stage 1 (leaf development) four to six true leaves in both fields from the BBCH extended scale [34].

For each study plot, a set of overlapped images (60% forward-longitudinal-lap and 30% side-lap) was captured from an MD4-1000 multi-rotor UAV (Microdrones GmbH, Siegen, Germany). The remote images were acquired using two different cameras, a conventional still visible camera, an Olympus PEN E-PMI (RGB, acquires 12-megapixel images in true colour, Red, R; Green, G; and Blue, B; image size 4032 × 3024 pixels, is equipped with a 14–42 mm zoom lens and sensor size 17.3 × 13 mm), and a multispectral camera, Tetracam mini-MCA-6 (TTC, 1.3-megapixel images in B (450 nm), G (530 nm), R (670 and 700 nm), R edge (740 nm) and near RS-Infrared (NIR, 780 nm); image size 1280 × 1024 pixels, focal length 9.6 mm and sensor size 6.66 × 5.32 mm). The aerial images were collected on 7 May 2014 at different flight altitudes (30, 60 and 100 m) using both cameras, although the flight at 60 m that used the RGB camera in Field 1 was not available because it was not correctly downloaded to the computer due to a processing problem. The flight lengths and areas flown and the associated altitudes and cameras are shown in Table 1.

The flight routes for each camera were programmed and automated, and only the take-off and landing were manually performed by the pilot. To create the geo-referenced ortho-mosaicked images, a total of six artificial GCPs were geo-referenced using a Trimble Geo-XH Differential GPS (DGPS) for each field. To construct the mosaicked image and facilitate the process, these GCPs had to be identified in all the images. The software used to process the images was Agisoft Photoscan Professional Edition (Agisoft LLC, St. Petersburg, Russia). Detailed information on the configuration of the UAV flights and specifications of the vehicle and cameras can be found in [21].

**Table 1:** Flight length and area flown over as affected by flight altitude and type of camera.

Field	Flight Altitude (m)	Sensor *			
		RGB		TTC	
		Length (min)	Area (ha)	Length (min)	Area (ha)
1	30	12	0.4	27	0.07
	60	- **	-		
	100	5	1.7	7	0.4
2	30	11.5	0.3	28	0.06
	60	5.4	0.6	11.1	0.14
	100	5	1.77	7	0.38

* RGB: Red-Green-Blue (visible range), TTC: Tetracam, Blue-Green-Red-Near Infrared range; ** data not available.

### 2.2. Resampling: Spatial Degradation of Fine Quality Images

In this study, the resulting ortho-mosaicked images for the 30 m flight altitude had a spatial resolution of 1.07 and 1.6 cm for the RGB and TTC, respectively, in both fields. The spatial resolution is defined automatically by the software that performs the mosaicking. This imagery was degraded through resampling to simulate pixel resolutions of 60 m (1.84 cm for the RGB and 3.25 cm for the TTC) and 100 m altitudes (3.07 and 5.42 cm for the RGB and TTC, respectively). Because the pixel size of the new RS-image is related to the altitude, focal length and resolution of the camera, the resampled pixel size has to be derived. Therefore, to obtain the corresponding pixel size, the nearest neighbour (NN) resampling method was used, which consists of the simplest reconstruction method whereby each pixel is assigned the intensity of the sample nearest to that pixel using ENVI software (ENVI 5.0, Research Systems Inc. Boulder, CO, USA, User Manual). This method does not modify the numerical value of the pixels, referred to as the digital number, and it is widely used because of the speed with which it can be implemented and its sheer simplicity [23,24]. The NN method simply chooses the pixel that has its centre nearest the point located in the image and this pixel is then transferred to the corresponding display grid location. This is the preferred technique if the new image is to be classified because it then consists of the original pixel brightness, simply rearranged in position to yield correct image geometry [35]. It is necessary to apply two factors, named xfactor and yfactor, which are calculated as the relationship between the pixel sizes of the image to the resample (*i.e.*, the image taken at 30 m altitude) divided by the resampled pixel targeted. A total of seven RS-images were created, three for Field 1 and four for Field 2 (two flight altitudes and two sensors). There are other resampling methods, e.g., bilinear and cubic convolution interpolations. The main difference compared with the NN method is that these methods do not preserve the original values because averages are used to obtain the digital number of the new pixels [23,36].

**Figure 1:**
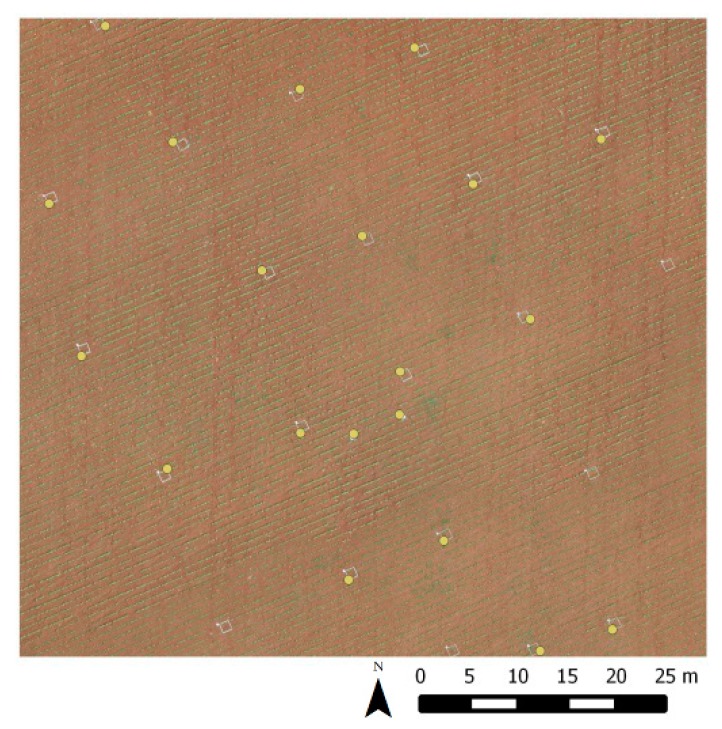
Representation of the 20 selected points for the American Society of Photogrammetry and Remote Sensing (ASPRS) test for Field 1.

Once the seven RS-images were created at 60 and 100 m flight altitudes for both cameras, and due to the availability of real UAV-images for these conditions, this latter imagery was used to establish a visual inspection and mathematical comparison of the quality of the spatial resolution and the spectral values of the output images. Spatial quality is usually judged by visual inspection; however, the human visual system is not equally sensitive to various types of distortion in an image. The spatial quality of the perceived image strongly depends on the observed scene as well as the viewing and the observer conditions [36]. To solve this matter, the positional accuracy of the RS-image was evaluated through a test of the American Society of Photogrammetry and Remote Sensing (ASPRS) [37]. This test establishes three quality classes according to horizontal accuracy defined by the root mean square error (RMSE) for a specific scale, where class 1 is the most precise and classes 2 and 3 are two and three times less precise, respectively. The RMSE is defined as the square root of the average of the squared discrepancies. A minimum of 20 points for each field that were easy to identify and distributed randomly over the entire field had to be selected from across the study areas to perform the test (Figure 1). The X and Y coordinates are evaluated separately and the one that had the worst results determined the quality of the RS-images. That is, ASPRS test defines horizontal accuracy classes in terms of their RSME X and Y values. Under the 1990 ASPRS standard, the allowable horizontal RMSE for Class 1 accuracy at 1:50 scale is 0.0125 m (1.25 cm). That represents a test condition that has to be accomplished to be classified in that class. In this work, the discrepancies are the differences in the coordinate values of the 20 selected points in the RS-image and the real UAV-image. Spectral values were also compared through information deduced from histograms to check that the NN method did not modify the digital number of the pixels.

### 2.3. Weed Detection

Once resampled, the following process was applied to the output RS-image. As a first step, a common area was delimited in both fields by creating a mask covering the area of interest (for the two cameras and the 60 and 100 m altitudes) to reduce the processing time. Subsequently, an OBIA procedure designed for the weed mapping tasks that was based on the weed mapping algorithm for maize crops in non-mosaicked imagery described in [33] was developed using the software eCognition Developer 8.9 (Trimble Geospacial, Munich, Germany). This algorithm was adapted and modified to this particular study according to the specific values of the sunflower fields. The OBIA algorithm developed was able to generate the weed maps, which provided information for site-specific weed control decision making. This algorithm consists of a three-step automatic classification approach: (1) image segmentation that defines vegetation and soil background objects; (2) discrimination of vegetation objects based on spectral information; and (3) classification of crop and weed plants based on the position of each plant relative to the crop-row structure.

The coincidence (or lack thereof) between the RS-image and UAV-image classifications was taken into account when the resampling accuracy was determined. Therefore, the ability of the OBIA algorithm to detect weeds in the RS-image was evaluated by studying the similarity of the results obtained from the real UAV-image equivalent under the simulated conditions (cameras, flight altitudes and fields). That is, the OBIA procedure was also applied to the ortho-mosaicked images created from the series of real UAV pictures acquired by the two sensors at 60 and 100 m altitudes in both fields.

Because the hypothesis that weed patch detection was based on the specification that every plant not located in the crop line was classified as a weed, the performance of the OBIA algorithm in every case study (camera, flight altitude and field) was evaluated by comparing the results obtained for crop-row identification and weed discrimination with observed real data obtained from 32 ground-truth 1 × 1 m (1 m^2^) sampling frames located in every field. These sampling areas were distributed across the entire study area and were representative of the weed infestation observed in the field and thus also included a number of weed-free sampling frames. The frames were visually divided into four categories of weed infestations ranging from 0 to 3 (eight samples in each category); 0 corresponded to no-presence (free of weeds), 1 corresponded to low infestation (approximately 30–50 pixels infested, around a 5% of area covered by weeds, corresponding to the image acquired at a 30 m flight altitude), 2 corresponded to medium infestation (approximately 75 pixels infested, around a 10%–15% of area), and 3 corresponded to high infestation (more than 100 pixels infested, around a 20% of area). To assess the accuracy of the results, a comparison of the area covered by weeds using all the frames in the RS-image *vs.* the UAV-image was established for each case. As stated previously, the term weed was applied to any type of vegetation that emerged between the crop lines. In addition, weed maps obtained using the OBIA procedure that were applied to both types of imagery (RS-image and UAV-image) were compared visually frame by frame. The objective was to determine whether weeds were detected or overlooked as a way to support the efficiency of the procedure in the RS-image.

Finally, an estimation of the weed-infested area was obtained and a strategy for SSWM was designed accordingly. This SSWM programme was based on the weed cover maps in which weed-free and weed-infested areas according to seven thresholds for every frame were considered. The thresholds assessed ranged from 0% (herbicide must be applied just when there is presence of weed) to 15% of the infested area (herbicide must be applied if weed coverage > 15%) with an increase ratio of 2.5. The frames were then classified as *Treatment* or *No-Treatment* depending on whether the threshold was exceeded or not. That is, seven herbicide treatment maps resulting from a given threshold value were studied for both the RS-image and the UAV-image at every flight altitude and for each camera.

## 3. Results and Discussion

### 3.1. Evaluation of Similarity and Quality between RS-Image and UAV-Image

The spatial resolution of the RS-image using the UAV-image acquired at 30 m and the UAV-image at 60 and 100 m are shown in Table 2. Taking into account that the pixel size of the UAV-image is directly related to the technical specifications of each camera and the flight altitude, the resampled pixel sizes were 1.84 cm and 3.25 cm at 60 m, and 3.07 and 5.42 cm at 100 m for the RGB and TTC cameras, respectively. Therefore, the new RS-image was downsampled and it has a lower file size than the UAV-image at 30 m due to pixel size increase and degradation of the image used as the baseline (*i.e.*, 150 MB for the UAV-image captured using the RGB at 30 m and 18.4 MB for the RS-image at 60 m for Field 1). The file size for the UAV-image at 30 m RGB and TTC are 150 MB and 122 MB for Field 1 and 237 MB and 237 MB for Field 2, respectively.

**Table 2:** Pixel resolution of UAV-image and RS-image, spatial quality (RMSE) and file size of RS-image according to flight altitudes and sensors in two sunflower fields.

Field	Sensor *	Flight Altitude (m)									
		60					100				
		Pixel Size (cm)		File Size (MB)	RMSE (cm)		Pixel Size (cm)		File Size (MB)	RMSE (cm)	
		UAV-I **	RS-I		X	Y	UAV-I	RS-I		X	Y
1	RGB	- ^§^	-	-	-	-	3.31	3.07	18.4	0.89	1.19
	TTC	3.2	3.25	13.3	1.22	1.12	5.41	5.42	4.82	1.13	1.21
2	RGB	1.99	1.84	75.8	0.87	0.67	3.37	3.07	27.2	1.24	1.11
	TTC	3.2	3.25	24.3	1.12	1.08	5.41	5.42	8.75	1.19	1.24

* RGB: Red-Green-Blue (visible range), TTC: Tetracam, Blue-Green-Red-Near Infrared range; ** UAV-I: UAV-imagery, RS-I: Resampled-imagery; ^§^ data not available.

The results of the spatial quality test according to the RMSE calculated from a total of 20 points for every sensor, flight altitude and coordinate are presented in Table 2. The RMSEs were similar regardless of the flight altitude and sensor used. They ranged from 0.87 to 1.24 cm and from 0.67 to 1.24 cm for the X and Y coordinates, respectively. The X coordinate showed the worst results and determined the quality of the RS-images. The RMSEs were lower than 1.25 cm for 1:50 scale, therefore the RS-images belonged to Class 1, which is the most precise class according to the ASPRS test conducted between the two types of imagery. The points selected for the ASPRS test in Field 1 are presented in Figure 1. It is likely that these results are accurate because the NN resampling method did not modify the digital values of the image, as expected. Moreover, the spectral values of the images and histograms from bare soil, crops and weeds for the UAV-image and the RS-image were analogous (spectral quality test). The information obtained from the histograms is shown in Table 3. The histograms were calculated from all the RS-images, but only those from the values from Field 2 and the RGB sensor are shown so as not to clutter Table 3. This similarity is very important for a further high matching of weed patch detection results from the RS-image *vs.* the UAV-image.

**Table 3:** Mean and Standard deviation of RGB-bands of UAV-image and RS-image created by Nearest-Neighbor resampling.

Band	UAV-I * 30 m	RS-I 60 m	RS-I 100 m
R	161.11 ± 24.25	161.11 ± 24.25	161.11 ± 24.25
G	121.64 ± 24.94	121.64 ± 24.93	121.64 ± 24.94
B	86.73 ± 22.98	86.73 ± 22.97	86.73 ± 22.98

* UAV-I: UAV-imagery, RS-I: Resampled-imagery.

### 3.2. Weed Detection: Mapping and Accuracy

**Figure 2:**
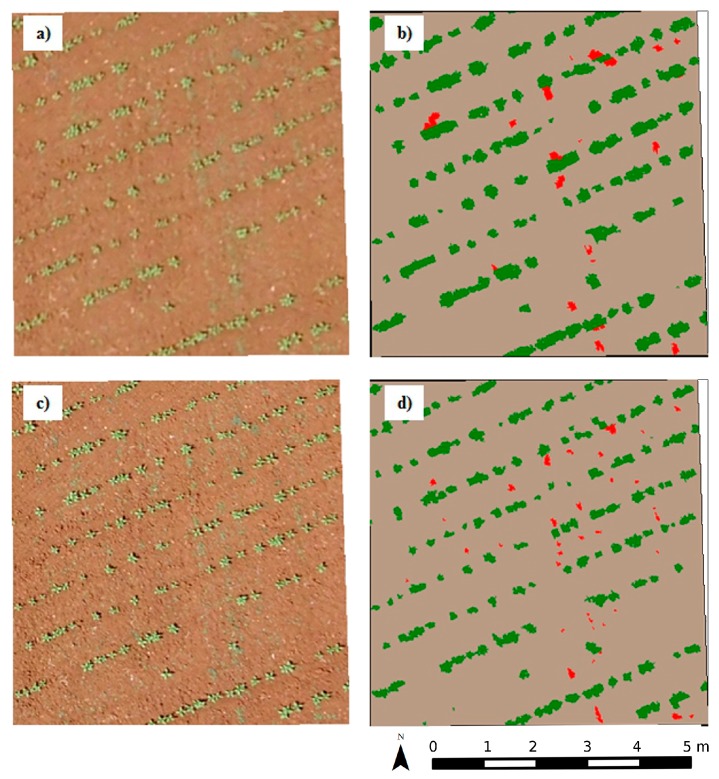
(**a**) Illustration of the UAV-image taken with the visible camera at 60 m; and (**b**) corresponding weed seedling map using object-based image analysis (OBIA) (green: crop rows, red: weed, grey: bare soil); (**c**) illustration of the RS-image at 60 m; and (**d**) corresponding weed seedling map using OBIA.

The OBIA procedure identified and mapped the sunflower crop rows with 95% accuracy in the RS-image and the UAV-image. Crop rows correctly identified and mapped are shown in Figure 2. This was due not only to the performance of the resampling procedure, but also to the high matching of the crop-line continuity of the ortho-imagery during the mosaicking process of the UAV-image used as a baseline. If these mosaics were not sufficiently accurate, the crop rows would appear interrupted or broken, and would be inaccurately geo-referenced and consequently, in an incorrect location, which would affect further resampling and classification [22]. Peña *et al.* [33] reported 100% accuracy in maize crops, although these authors analysed non-mosaicked UAV-images, *i.e.*, the UAV images were studied one by one.

**Figure 3:**
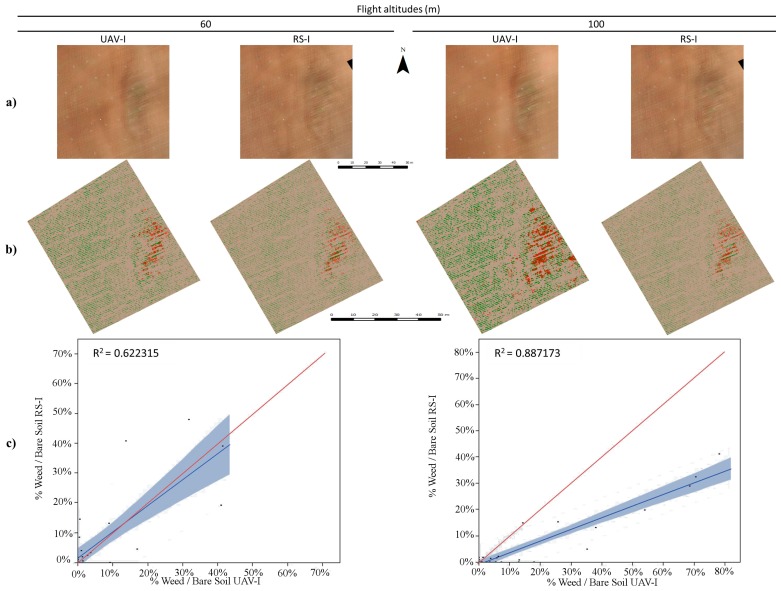
(**a**) Portions of UAV-image taken with the visible camera and RS-image; (**b**) maps obtained using OBIA; and (**c**) graphics comparing UAV-image and RS-image estimated weed cover in the 32 frames established in Field 2.

**Figure 4:**
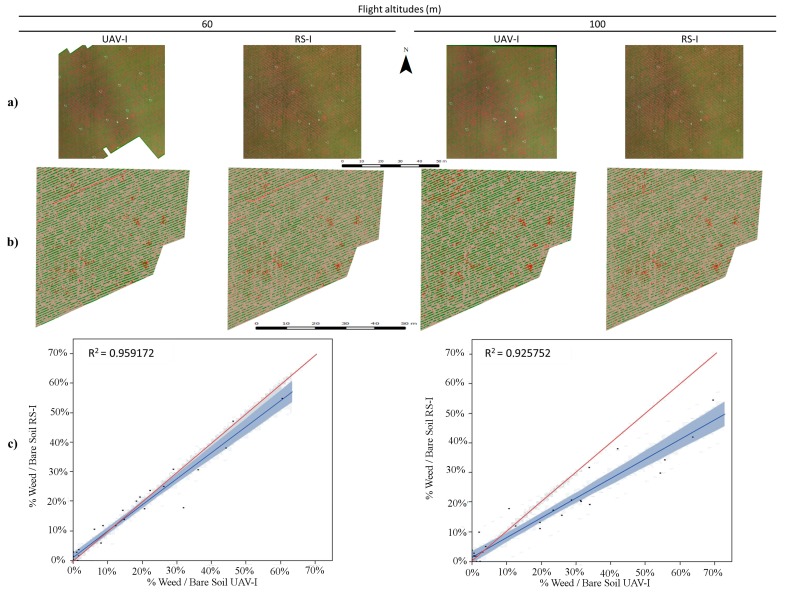
(**a**) Portions of UAV-image taken with the multispectral camera and RS-image; (**b**) maps obtained using OBIA; and (**c**) graphics comparing UAV-image and RS-image estimated weed cover in the 32 frames established in Field 1.

Therefore, our results surpass these because they are able to offer a crop-row map of the whole study area. Other authors have reported difficulties in obtaining ortho-mosaicked imagery from row crops such as maize, even when their objective was to determine the effect of topography on the rate of cross-pollination, *i.e.*, they did not map the crop rows [38]. Hence, the excellent results obtained for crop-row detection and mapping were related to the robustness of the mosaicking of the 30 m flight used as a baseline together with the resampling procedure and the OBIA methods developed. This has strong implications for the success of the next step, *i.e.*, the detection of weeds located in the areas between the rows. To evaluate the accuracy of this classification process, a comparison of the weed coverage in 32 frames was performed between the RS-image and the UAV-image for both fields and sensors. Field 1, using the TTC camera, and Field 2, using the RGB camera, are shown in Figure 3 and Figure 4, respectively. The percentages of the weed patches in the RS-image were close to those obtained in the UAV-image; this was supported by the narrow width of the coincidence of the fitting line, and the coefficient of determination was also evaluated. In the case of the RGB camera in Field 2 (Figure 3c), there was a slightly higher variation and lower fitting between the two types of images (RS-image and UAV-image).

As a first result, the percentage of the weed coverage estimated for both types of sensors and altitudes in the RS-image and the UAV-image is shown in Table 4. In all cases, except for the images from the RGB camera at an altitude of 100 m, resampling tended to detect more weed cover and consequently the possible site-specific area to treat would be larger. From an agronomic point of view, the over-estimation of weeds for generating weed maps is more acceptable than non-detection or under-estimation. Farmers would choose to treat weed-free areas rather than assume the risk of allowing weeds to go untreated [14,39]. Even if the area covered by weeds to be treated differed between RS-images and UAV-images, an important reduction in herbicide would be reached compared with the traditional management, which would consist of herbicide application over the entire field. The observed differences could be due to variations in the quality in terms of the spectral information (*i.e.*, the RS-image pixels at 60 m were resampled, preserving the original spectral values from the UAV-image at 30 m; the pixel sizes were 1.07 cm for RGB and 1.6 cm for TTC. In contrast, the UAV-image pixels at 60 m could represent a spectral mixture due to a greater pixel size at that altitude, *i.e.*, pixel sizes were 2 cm and 3.2 cm for RGB and TTC, respectively) or variation in the performance of the OBIA algorithm, which may have detected more weed cover in the RS-image compared with the UAV-image in some cases.

**Table 4:** Percentage of area covered by weed estimated.

Field	Sensor	Flight Altitude (m)			
		60		100	
		Estimation of Area Covered by Weed (%)			
		UAV-I *	RS-I	UAV-I	RS-I
1	RGB	- **	-	23	17
	TTC	25	40	29	46
2	RGB	10	12	20	12
	TTC	16	19	20	21

* UAV-I: UAV-imagery, RS-I: Resampled-imagery; ** data not available.

**Figure 5:**
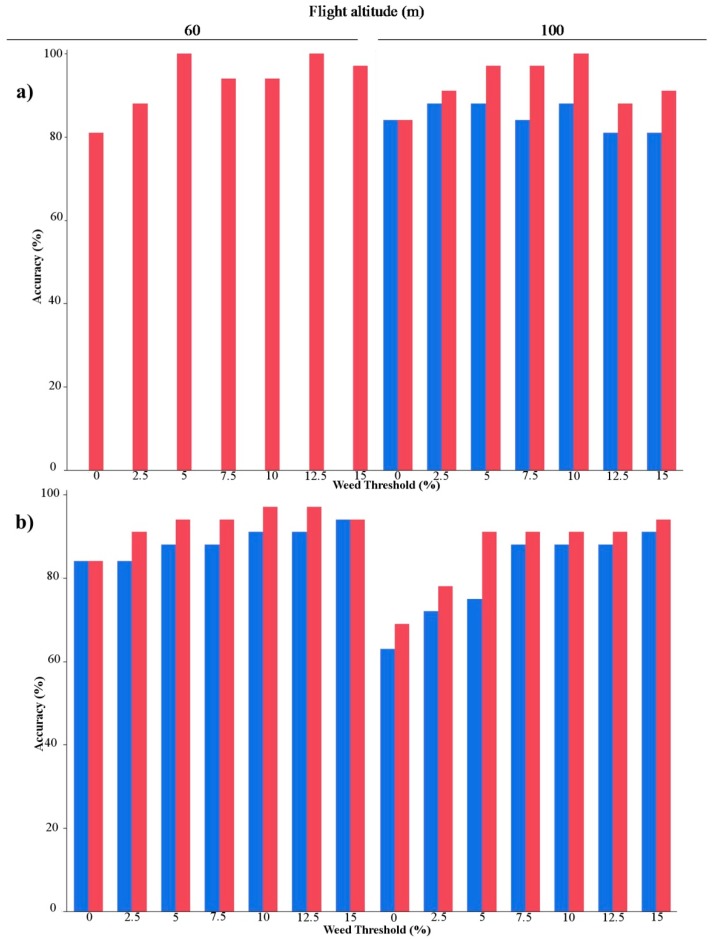
Accuracy (%) of match in the classification (*Treatment* or *No-Treatment*) of the 32 frames according to seven weed thresholds for the RS-image with the still visible and multispectral cameras (blue: RGB; red: TTC) at 60 m and 100 m altitude for (**a**) Field 1; (**b**) Field 2.

The percentage matching in the weed-patch and weed-free discrimination areas based on the *Treatment* or *No-Treatment* classification of the 32 frames as affected by flight altitude and camera using the seven threshold values established is shown in Figure 5 for both sunflower fields. The algorithm for weed detection performed better for the RS-image created from imagery captured using the multispectral camera (TTC). The best results were obtained for the 60 m altitude, and 100% coincidence in the classification was reached for the 5% and 12.5% weed thresholds, although a slight decrease can be observed for the other cases. Almost all of the cases followed the same trend, *i.e.*, the percentage accuracy improved with an increase in the weed thresholds. A possible explanation for these results is that the OBIA algorithm easily detects larger weed patches compared with small ones, because of the low number of pixels corresponding to weeds. In that case, there are not enough weed pixels to form the weed objects, and these objects are not correctly built and consequently present a mixture of bare soil and weeds. Then, the OBIA is not able to detect the spectral differences between the weeds and bare soil, which reduces the performance of the algorithm. The representation of the percentage match in both fields is shown in Figure 5. The values of the different thresholds established as a decision making tool could vary depending on the crop and its ability to compete for resources compared with the ability of the weeds [11].

**Figure 6:**
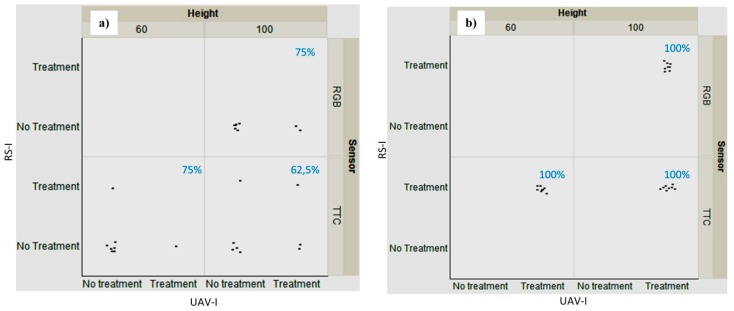
Representation of concordance of *Treatment* and *No-Treatment* for every eight frames corresponding to the weed infestation level category 0 and 3 infestation level (**a** and **b**, respectively) for RS-image and UAV-image at 60 and 100 m altitudes, both types of sensors in Field1. Abscissa and ordinate axis correspond to UAV-image and RS-image classification respectively. Percentage of concordance in every case is shown in blue.

The agreement or disagreement in the classification of *Treatment* and *No-Treatment* for the eight frames corresponding to the 0 and 3 infestation levels for the RS-image and the UAV-image for both sensors and altitudes in Field 1 is illustrated in Figure 6. Each of the eight points of every quadrant corresponds to the frames of its infestation category. A higher classification matching for any of the quadrants was obtained when the eight frames of every infestation category coincided with the corresponding *Treatment* or *No-Treatment* approach for the RS-image and the UAV-image. A poorer classification was recorded when the eight frames were dispersed between *Treatment* and *No-Treatment*. In Field 1, there was a total (100%) coincidence in the classification for categories 2 (medium infestation, data not shown) and 3 (high infestation, Figure 6b), which was independent of the sensor and altitude considered. This can be observed in the figure where the cloud of points matched their corresponding treated and untreated areas. The two other categories, no-presence (category 0, Figure 6a) and low infestation (category 1, data not shown) exhibited a lower agreement, showing that 71% and 63% of the frames matched, respectively.

In Field 2, 100% accuracy was reached for category 3, whereas 72%, 72% and 63% of the frames showed concordance for categories 0, 1 and 2, respectively. Generally, slightly better results were obtained at 60 m for both fields. From a weed control point of view, the critical infestation that can affect the sunflower yield corresponds to the medium and high infestation categories [8]; therefore, an agreement for *Treatment* and *No-Treatment* for all the frames involved is highly desired when comparing the results for the RS-image and the UAV-image. There is no doubt that farmers have to treat those areas where weeds emerge at high densities because competition with sunflower for available resources is more relevant.

**Figure 7:**
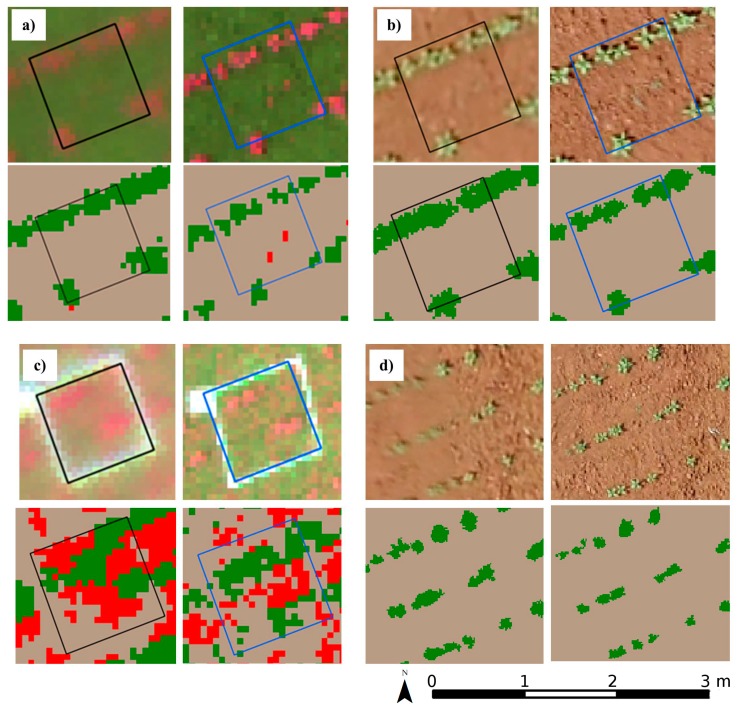
Examples of events that occurred in the classification of frames between UAV-image (left) and RS-image (right) for both sensors. Upper and bottom figures display the ground truth data of corresponding squared frames and classified image, respectively. Sunflower crop rows and weeds are represented in green and red color, respectively. (**a**) UAV-image using TTC at 100 m altitude and RS-image at 100 m (weed is detected only in the RS-image); (**b**) UAV-image using RGB at 60 m altitude and RS-image at 60 m (weed is not detected in any case); (**c**) UAV-image using TTC at 100 m and RS-image at 100 m (mixture between line crop and weed pixels); and (**d**) UAV-image using RGB at 60 m altitude and RS-image at 60 m (distortion in the UAV-image avoids a correct crop shape definition).

Some of the possible cases that can occur in the classification of the RS-image and the UAV-image are shown in Figure 7. As an example, Figure 7a illustrates the difference in the classifications when weeds were present (ground-truth data); however, they were only detected in the frame of the RS-image at 100 m, but not in the UAV-image acquired at 100 m. Consequently, this classification would have a poorer match, even though the OBIA procedure worked correctly in the RS-image. One of the disagreements in the detection of weeds for the low infestation frames in both the RS-image and the UAV-image (underestimation of weed cover) is displayed in Figure 7b, indicating that in this case the classification matched (low infestation was not detected in any of the imagery) although the OBIA procedure operated with certain limitations and was not able to detect this small infestation. Another misclassification was observed in the areas affected by an extremely high weed infestation (Figure 7c), where objects corresponding to weeds and the crop were mixed together and the OBIA procedure was not able to perfectly distinguish crop lines. This type of disagreement has less relevance than the others because this field area will likely be included in a large weed-infested area that would be easily mapped without many difficulties. In addition, when distortions or loss of sharpness are present in the UAV-image used as the baseline for the resampling, these will also be apparent in the RS-image. Conversely, if the UAV-image appears sharper, then the RS-image quality is also visually better. This can be observed in the upper and right side illustrations of Figure 7d.

The results obtained were satisfactory and will allow the optimization of high overlapping and low altitude UAV flights that require early weed patch mapping. Both parameters are crucial for generating very high spatial resolution imagery, although they increase the computational costs, human resources and the time consumed in mosaicking. This inconvenience could be solved by resampling from low to high altitude flights to improve the efficiency of the whole methodology. For example, the application of OBIA to the study area using the UAV-image at a 30 m altitude took approximately 90 min compared with 15 min (85% less) for the RS-image at a 100 m altitude; here, the accuracy was maintained but there was a smaller number of pixels in the RS-image. According to our results, the spatial resolution of the RS-image (3.07 and 5.42 cm for the RGB and TTC cameras, respectively) would still be suitable for the accurate detection of weed patches at an early growth stage. The horizontal accuracy (RMSEs) for the RS-image at 60 and 100 m ranged between 0.87 and 1.24 cm, which are acceptable for assessing high quality images according to the 1990 ASPRS test. These results together with the fully automated OBIA methodology show the clear advantage of resampling because using an image from a low altitude flight and degrading its spatial resolution makes it possible to obtain an accurate RS-image for studying weed patch detection.

Weed patch mapping resulting from imagery acquired from a UAV flying at an altitude of 100 m is quite similar to the RS-image at this altitude. However, our suggestion for the scientific community and general users interested in weed control decision making would be to not fly at 100 m (even if a greater field area is covered), but preferably to resample a UAV-image acquired at a lower altitude with a high spatial resolution to obtain the RS-image with the corresponding pixel size. The reasons for this recommendation are: (1) the incidences attributable to wind (even if it is lower than 6 km·h^−1^, which is the maximum limit for the UAV used in this research) are more noticeable at higher altitudes due to distortions and lack of sharpness similar to those observed in Figure 7d. These problems are much less evident flying at a 30 m altitude and consequently they do not appear in the RS-image; (2) although the RS-image at a 100 m altitude has a spatial resolution (pixel size) similar to the UAV-image at that altitude, the RS-image maintains the high spectral information of the UAV-image at a 30 m altitude used as the baseline for the resampling (as observed in Table 3); and (3) this high quality spectral information is crucial for a better performance of the OBIA algorithm for early weed patch detection in the RS-image (Figure 7a). That is, when the size of any weed seedling (e.g., approximately 3 cm) is lower than the pixel size of the UAV-image acquired at a 100 m altitude (around 5.4 cm), a mixture of bare soil and weeds is present in that pixel and the creation of weed objects may be inadequate. Even when the pixels of this imagery also have a 5 cm size, this is less evident in the RS-image at 100 m because they originated from the UAV-image at 30 m with 1.07 cm pixels, which would correspond to pure weed pixels (*i.e.*, a weed seedling of 3 cm would cover 3 pixels) and this would favour both the better sharpness of the RS-image and the performance of the classification algorithm.

The differences observed could be tolerable as the resampling tended to over-estimate weed cover, and farmers usually prefer a conservative option and treat a greater area than needed to ensure crop development. These results are also essential for providing accurate information to a SSWM plan, because it is feasible to establish different zones that are adequate for a site-specific weed control strategy. This is relevant not only for reducing herbicide use, but also for optimizing energy (fuel) and field operating time and money. This is because there are areas where the equipment used to spray herbicide would not enter at any time as the lack of weed emergence, independent of the weed threshold applied.

Slightly better results were obtained for the TTC. This is important because this sensor presents more limitations related to the area covered and flight length due a higher number of images required to cover the study area compared with the RGB camera. This means that ortho-mosaicking of UAV imagery could present some computational limitations due to the requirements involved in the stitching of the numerous sets of images. These problems could be solved by accurately simulating what will occur at higher altitudes using resampling, therefore reducing time processing.

Another possible advantage of using the RS-image is when a set of different flight altitudes are tested in a study and any incidence occurs due to the UAV-battery limitations or the worsening of weather conditions. Our results show that the experiment could be completed if resampling is applied to the available UAV imagery acquired at lower altitudes. An additional possibility for using resampling may be related to the optimization of tests in a preliminary study in which it is necessary to extrapolate the results to a larger surface. That is, if the results obtained from an RS-image at 100 m are accurate and satisfactory, they could be very useful for the decision making process of capturing new images from 100 m to try to expand the analysis and increase the extent of the surface flown over. If these preliminary tests are not convincing, it is not worth planning further flights. Regarding the computation costs and the improvements made when using the RS-image, it was shown that the application of the image analysis procedures took less time for the RS-image compared with the original UAV-image acquired at a 30 m altitude due to the RS-image having a lower spatial resolution than the UAV-image and a much lower number of pixels. This will have a considerable influence on the efficiency of the image analysis (time processing).

## 4. Conclusions

The results obtained support the use of resampling in our study cases. This study shows an NN resampling procedure to extract the digital spectral values from high spatial resolution imagery as an alternative methodology for optimizing the limitations usually present in the UAV imagery. To the best of our knowledge, no quantitative evaluation of RS-image ortho-mosaicked quality from UAV-images for early weed detection and mapping using OBIA has been reported or published so far. The assessment was performed to evaluate the image quality and the spatial resolution of the RS-image on the early weed discrimination for two cameras and in two fields demonstrated the consistence of our results when the pixel resolution was within the range of 3 to 5 cm. These results are useful to enhance the current advantage of the UAV because accurate weed cover maps were generated from the degraded spatial resolution of the RS-image data at 60 and 100 m compared with the UAV-image from real flights at those altitudes. This has many potential applications in other agronomic tasks in which a timely and fine resolution map must be produced.
